# Behavioral lateralization of mice varying in serotonin transporter genotype

**DOI:** 10.3389/fnbeh.2022.1095567

**Published:** 2023-01-11

**Authors:** Binia Stieger, Yvonne Wesseler, Sylvia Kaiser, Norbert Sachser, S. Helene Richter

**Affiliations:** ^1^Department of Behavioural Biology, University of Münster, Münster, Germany; ^2^DFG Research Training Group EvoPAD, University of Münster, Münster, Germany

**Keywords:** anxiety, behavioral lateralization, depression, handedness, mice, psychiatric disorders, serotonin transporter (5-HTT)

## Abstract

In humans, non-right-handedness is associated with a higher incidence of psychiatric disorders. Since serotonin seems to be involved in both, the development of psychiatric disorders and lateralization, the present study focuses on the effect of the serotonin transporter (5-HTT) gene on behavioral lateralization. For this, we used the 5-HTT knockout mouse model, a well-established animal model for the study of human depression and anxiety disorders. For female mice from all three 5-HTT genotypes (wild type, heterozygous, and homozygous knockout), we repeatedly observed the direction and strength of lateralization of the following four behaviors: grid climbing (GC), food-reaching in an artificial test situation (FRT), self-grooming (SG), and barrier crossing (BC), with the FRT being the standard test for assessing behavioral lateralization in mice. We found no association between behavioral lateralization and 5-HTT genotype. However, in accordance with previous findings, the strength and temporal consistency of lateralization differed between the four behaviors observed. In conclusion, since the 5-HTT genotype did not affect behavioral lateralization in mice, more research on other factors connected with behavioral lateralization and the development of symptoms of psychiatric disorders, such as environmental influences, is needed.

## 1. Introduction

Handedness is asymmetrically distributed in the human population, with about 10% of all people being left-handed (Papadatou-Pastou et al., 2020). The differential expression of handedness has been linked to a range of psychiatric disorders (Mundorf and Ocklenburg, 2021). To be more specific, an increased prevalence of non- right-handedness was found among individuals with schizophrenia (Sommer et al., 2001; Hirnstein and Hugdahl, 2014; Ravichandran et al., 2017), bipolar disorder (Ravichandran et al., 2017), and autism (Markou et al., 2017). Mixed results exist for major depressive disorder (MDD) and anxiety disorders. For MDD, there are several studies suggesting an association between non-right-handedness and the disorder (Bruder et al., 1989; Beiderman et al., 1994; Denny, 2009; Logue et al., 2015), whereas a recent meta-analysis does not support these findings (Packheiser et al., 2021). Regarding anxiety disorders, an association with non-right-handedness could be found only in children (Logue et al., 2015) and individuals with inconsistent handedness (Lyle et al., 2013). However, not only behavioral lateralization, but also cerebral lateralization is linked with different neurodevelopmental and psychiatric disorders (Mundorf et al., 2021; Kong et al., 2022). With the exception of MDD, a range of disorders were associated with distinct patterns of structural hemispheric differences (Mundorf et al., 2021; Kong et al., 2022). It is suggested that these distinct patterns of alterations are related to cognitive functions that are associated with the symptomology of the respective disorders (Mundorf et al., 2021).

In humans, an important risk factor for the development of some of the most frequent psychiatric disorders is the serotonin transporter (5-HTT) gene (*SLC6A4*). More precisely, a repeat length polymorphism in the transcriptional control region of the 5-HTT gene leads to either a short or long allele. The short allele produces significantly less 5-HTT than the long. Since 5-HTT reaccumulates released serotonin into the presynaptic neuron, carrying the short allele results in higher concentrations of serotonin in the synaptic cleft (Canli and Lesch, 2007). And, carrying the short allele increases the risk of suffering from depression and anxiety disorders (Lesch et al., 1996; Canli and Lesch, 2007). Thus, the encoded 5-HTT variation can lead to psychiatric disorders associated with serotonin dysregulation.

Besides serotonin dysregulation, neurotransmitters in general seem to be important for lateralization and susceptibility for symptoms of psychiatric disorders. In rodents, relative right hemisphere dopamine content is associated with the direction and strength of paw preference (Schwarting et al., 1987; Cabib et al., 1995; Nielsen et al., 1997). Furthermore, an increase of serotonin turnover in only the left hemisphere after an immune challenge was solely observed in ambilateral and right-pawed, but not left-pawed mice (Delrue et al., 1994). Lastly, asymmetrical contents of serotonin and dopamine in the hemispheres have been associated with symptoms of depression and anxiety in rats. For example, a higher content of serotonin and dopamine in (specific areas of) the right versus the left hemisphere correlates with increased anxiety (Andersen and Teicher, 1999). However, due to the partly contradictory findings for the link between the psychiatric disorders MDD and anxiety disorders and lateralization in humans, and lacking studies on the role of serotonin in lateralization, further studies are needed to clarify this association. Against this background, the current paper focuses on the link between the 5-HTT gene, a risk factor for the development of psychiatric disorders and a regulator of serotonin brain content, and behavioral lateralization.

To study the role of the 5-HTT gene in behavioral lateralization, we applied the well-established 5-HTT knockout mouse model for human psychiatric disorders. This model benefits from a targeted disruption of the 5-HTT gene, that leads to increased amounts of depression- and anxiety-like behaviors in 5-HTT heterozygous (+/−) and homozygous (−/−) knockout, compared to wild type (+/+) mice (Bengel et al., 1998; Holmes et al., 2003; Lira et al., 2003; Zhao et al., 2006; Kalueff et al., 2007; Popa et al., 2008; Heiming et al., 2009; Lewejohann et al., 2010; Krakenberg et al., 2019). Furthermore, mice are an ideal study system as they have been shown to exhibit measurable paw and side preferences (Collins, 1968; Manns et al., 2021). About 80% of individuals show a significant paw preference on the individual level. However, a population level asymmetry is absent (Manns et al., 2021). The standard method to assess behavioral lateralization in mice, and rodents in general, is a food-reaching test (FRT) (e.g., Manns et al., 2021), introduced by Collins (1985). Using this method, it has been found that, similar to human handedness, preferences in mice can be temporally stable (Collins, 1985; Betancur et al., 1991; Stieger et al., 2021), different for separate tasks (Collins, 1975; Betancur et al., 1991; Signore et al., 1991; Waters and Denenberg, 1994; Biddle and Eales, 1996; Nielsen et al., 1997; Stieger et al., 2021) and sex dependent (Collins, 1975; Betancur et al., 1991). In a previous study (Stieger et al., 2021), we studied behavioral lateralization for four different behaviors of mice from two different strains and both sexes. We applied the standard method (FRT) and observed three more spontaneous behaviors (equivalent to the behaviors in the present study, see below). Relevant in this context were the findings that individuals were more strongly lateralized for the FRT compared to the three spontaneous behaviors. Additionally, directional side preferences were temporally stable for all behaviors but only the strength of preferences in the FRT was stable over time. Lastly, preferences in the spontaneous behaviors were unrelated to those from the FRT (Stieger et al., 2021).

Against this background, we employed female mice from the 5-HTT knockout model and assessed their behavioral lateralization. More precisely, we measured the direction and strength of behavioral lateralization of mice from all three 5-HTT genotypes: +/+, +/−, and −/−. Since both, the direction and strength of lateralization can be time- (e.g., Betancur et al., 1991; Stieger et al., 2021) and task dependent (e.g., Waters and Denenberg, 1994; Stieger et al., 2021), we assessed temporal and task consistency for the different behaviors. To do this, we repeatedly observed the following four behaviors: grid climbing (GC), FRT, self-grooming (SG), and barrier crossing (BC). We hypothesized to find differences in behavioral lateralization across the three genotypes. Furthermore, in line with previous studies, we expected to find time- and task-dependent differences in behavioral lateralization (Betancur et al., 1991; Waters and Denenberg, 1994; Stieger et al., 2021).

## 2. Animals, materials and methods

### 2.1. Animals and housing conditions

In this study, female mice from a serotonin transporter (5-HTT) knockout model (Bengel et al., 1998), backcrossed into a C57BL/6J genetic background for more than 10 generations, were used. We included wild type (+/+; *N* = 18), heterozygous (+/−; *N* = 18) and homozygous knockout mice (−/−; *N* = 12). Sample sizes differ due to different breeding successes of the individual genotypes. The animals originated from the internal breeding stock of the Department of Behavioural Biology at the University of Münster, Germany. The original heterozygous breeding pairs were provided by the Department of Molecular Psychiatry at the University of Würzburg, Germany. For genotyping, genomic DNA was extracted from ear tissue and amplified by PCR. Genotypes were identified by agarose gel electrophoresis of DNA fragments with lengths of 225 bp (5-HTT +/+), 272 bp (5-HTT −/−) or both (5-HTT +/−). After weaning, mice were housed in groups of 2–5 animals per cage. They were marked with ear cuts to allow for individual identification. Approximately 3 weeks before the experiment started, group housing was changed to pair housing, with two mice of different genotypes. At the beginning of the experiment, mice were between 104 and 324 days old. Animals were kept in standard Makrolon cages type III (37 cm × 21 cm and 15 cm high) with wood shavings as bedding material (Tierwohl, J. Reckhorn GmbH & Co., KG, Rosenberg, Germany). The cages were enriched with a semitransparent red plastic house (Mouse House™, 11.1 cm × 11.1 cm and 5.5 cm high, Tecniplast Deutschland GmbH, Hohenpeißenberg, Germany), a wooden stick (ca. 10 cm × 1.8 cm and 1.8 cm high) and a paper towel as nesting material. Food (Altromin 1324, Altromin GmbH, Lage, Germany) and tap water were provided *ad libitum*, except for the time directly before the FRT, when the food was removed. Cages were changed and a new paper tissue was provided on a weekly basis, whereas the plastic houses and wooden sticks were renewed every 2 weeks. The housing room was kept at a reversed 12 h dark/light cycle with lights off at 0900 h, an ambient temperature of about 22°C and a relative air humidity of about 50%.

### 2.2. Experimental design

**FIGURE 1:**
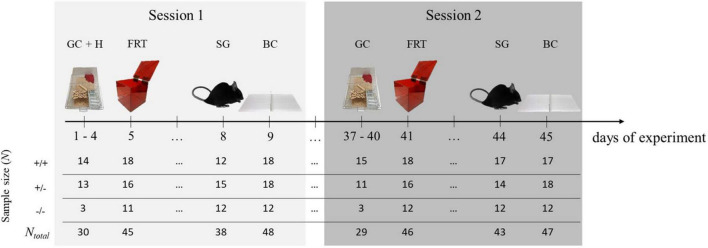
Experimental design. To determine behavioral lateralization, four behaviors [grid climbing (GC), food-reaching (FRT), self-grooming (SG), and barrier crossing (BC)] were observed. Additionally, in session 1, there was a 4-day lasting habituation phase for the test box (H). To test for the temporal consistency of behavioral lateralization, mice underwent the same observations 5 weeks later. In each session, the behavior of each mouse was observed only once. At the beginning of session 1, animals were between 104 and 324 day old. At the beginning of session 2, they were between 141 and 361 days old. Sample sizes differed between the behaviors and sessions because not all mice reached the minimal number of counts per behavior (see the following sections) and therefore had to be excluded from the statistical analyses. +/+ = 5-HTT wild type mice, +/− = 5-HTT heterozygous knockout mice, −/− = 5-HTT homozygous knockout mice.

The experimental design applied here was similar to the one described previously (Stieger et al., 2021). Briefly, in a first testing session [PND’s (postnatal days) at beginning of session: 104–324; adulthood], the lateralization of four different behaviors was observed (GC, FRT, SG, BC). All observations were conducted within nine consecutive days in the order stated. For the FRT, we included a habituation phase to the test box during the first 4 days of the experiment. To test for temporal consistency of behavioral lateralization, mice underwent the same observations 5 weeks later (session 2; PND’s at beginning of session: 141–361, adulthood). The sequence of the observed behaviors was kept the same for both sessions in order to ensure good comparability over the two time points. In the second session, however, no habituation phase to the test box was scheduled (Figure 1).

The experiment was conducted in two batches by two experimenters. Half of the data (*N*_+/+_ = 8, *N*_+/–_ = 8, *N*_–/–_ = 8) was obtained in a first batch by YW, and the other half in a second batch (*N*_+/+_ = 10, *N*_+/–_ = 10, *N*_–/–_ = 4) by BS. Because of likely batch- and experimenter-induced variation, batch was systematically integrated as a controlled variable (Von Kortzfleisch et al., 2020) and was accounted for in the models for the statistical analysis.

### 2.3. Behavioral observations

Behavioral observations were performed and video recorded during the animals’ active phase between 0900 and 1600 h, i.e., during the dark phase of the light cycle in the animals’ housing room. The order of the observed behaviors was the same for all animals (see Figure 1). However, the order of the mice observed on the single days was randomized. The experimenter was blind to the animals’ genotypes during the behavioral observations and video analysis.

#### 2.3.1. Grid climbing

**FIGURE 2:**
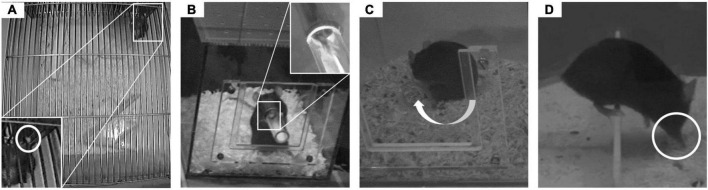
Behavioral observations. In the experiment, four behaviors were observed. **(A)** The paw that was used for commencing grid climbing (GC) was observed. **(B)** In the food-reaching test (FRT), the paw used to reach for food in the feeding tube was counted. **(C)** The turning side for self-grooming (SG) the tail was noted. **(D)** Whilst crossing a barrier (BC), the paw that was used by mice to descend the barrier was counted.

Climbing activity is a major component of mouse behavior in standard laboratory environments. To commence climbing, mice reach for and grab a cage grid with one forepaw (Figure 2A). Climbing in the home cage was recorded from an aerial view (cameras: 1,000 H Nano, AVerTM Information Europe B.V., Rotterdam, Netherlands) for 5 h during the active phase. For an easier identification of the animals from this top down view, their tails were marked with a black pen at different positions. The recordings were analyzed by hand.

#### 2.3.2. Food-reaching test

Paw preference of mice for reaching for food was assessed using an established method (Collins, 1968) with minor modifications. The cubic test box (14 cm × 14 cm × 14 cm high) was made out of red, semi-transparent plastic. A cylindrical, removable feeding tube (9 mm diameter) could be attached to the front wall in an equidistant position from the two sidewalls. To habituate the animals to the test box (Ribeiro-Carvalho et al., 2010), they were repeatedly exposed to this new environment. For detailed information, please refer to (Stieger et al., 2021). In preparation of the test, the food in the animals’ home cages was removed. For testing, mice were individually placed in the test box. After 5 min of acclimatization, the feeding tube was attached and animals were required to perform a reaching task in order to retrieve mash-like food (dissolved baby oat flakes) from the tube (Figure 2B). A camera (SONY HDR-XC6, with night shot mode) recorded the paw reaches for 15 min. Recordings were analyzed manually using the freeware behavior coding program Solomon Coder (version: beta 17.03.22). After coding 50 reaches, the analysis was terminated [e.g., (Collins, 1968; Waters and Denenberg, 1994; Fu et al., 2003)].

#### 2.3.3. Self-grooming

Self-grooming was observed in the same test boxes as mentioned above. To enhance self-grooming rates, mash-like food (dissolved baby oat flakes) was applied on the tail and lower back of the animals. A camera (SONY HDR-CX6, with night shot mode) recorded the grooming behavior for 15 min. Recordings were analyzed manually with Solomon Coder (version: beta 17.03.22) to assess the animals’ turning side preference for grooming their tail (Figure 2C).

#### 2.3.4. Barrier crossing

Barrier crossings were observed in a modified standard housing cage (Macrolon cage type III), that was divided in half by a transparent plastic barrier (30 mm high). A camera (SONY HDR-XC6, with night shot mode) recorded the behavior for 15 min. Recordings were used to assess paw preference performance by observing the forepaw used by the mice to climb down the barrier (Figure 2D). Solomon Coder (version: beta 17.03.22) was used to manually analyze the videos. After coding 50 crossings, the analysis was terminated.

### 2.4. Statistical analysis

Data was analyzed using the statistical software R (R Core Team, 2020, Version 4.0.3) and R Studio (RStudio Team, 2020, Version 1.3.1093). In cases where we calculated linear mixed effects models, we graphically examined and tested their residuals for normality and homoscedasticity using the Shapiro–Wilk test. We transformed raw data to meet the model assumption of normally distributed model residuals (see Supplementary Tables 1, 2 for more details). If interactions and main effects were significant, Tukey HSD *post-hoc* comparisons were conducted. Partial eta squared (η^2^p) was calculated as a measure of the magnitude of the reported effects (Lakens, 2013). Differences were considered to be significant at *P* ≤ 0.05.

#### 2.4.1. Data preparation

The first steps of the approach to the statistical analysis applied here were the same as described previously (Stieger et al., 2021). Basically, it was recorded how often the animals used their right or left paw to start climbing, remove food, cross a barrier, or, to which side they turned for grooming their tail. Only data from animals that reached at least 10 counts per behavior and session were included in the statistical analysis.

To evaluate whether a mouse had a significant side preference (left or right) or not (ambilateral), a binomial Z-score was calculated using the following formula:


$$Z-score=\frac{\text{R}-\frac{\text{N}}{2}}{\sqrt{\text{N}\times \text{p}\times \text{q}}}$$

where R is the number of right side preferences, N the total number of counts, and *p* = *q* = 0.5. Mice with Z-scores higher than 1.96 were considered rightward lateralized, those having Z-scores lower than −1.96 were considered leftward lateralized, whereas animals having Z-scores in between were considered ambilateral (see e.g., Dodson et al., 1992; Wells, 2003).

Additionally, for each animal, a handedness index (LI) was calculated to evaluate the direction of side preferences (e.g., Hopkins and de Waal, 1995; Wells, 2003) using the following formula:


$$LI=\frac{\text{R-L}}{\text{N}}$$

where R is the number of right side preferences, L the number of left side preferences and N the total number of counts. A LI of -1 depicts a strong left side preference, whereas a LI of 1 reflects a strong right side preference.

Lastly, absolute values of LI (|LI|) were used to evaluate the strength (magnitude) of laterality independent of the direction of side preference (e.g., Hopkins and de Waal, 1995; Wells, 2003). A |LI| of 0 indicates no preference for either side, whereas 1 depicts a strong preference for one side.

#### 2.4.2. Assessment of lateralization of behaviors

To assess whether lateral biases across the three genotypes deviated from a random distribution, we calculated chi^2^-tests with the number of right side preferring (R), left side preferring (L) and ambilateral (A) mice for each behavior and genotype in session 1. We compared the respective numbers for each behavior separately. For those behaviors and genotypes where this distribution deviated from randomness, we further conducted the following pairwise comparisons using binomial tests: right side preferring (R) vs. left side preferring (L), right side preferring (R) vs. ambilateral (A) and left side preferring (L) vs. ambilateral (A). Where relevant, *P*-values were adjusted for multiple testing using Bonferroni correction.

#### 2.4.3. Effect of genotype on direction and strength of lateralization

One model was fitted to analyze the effect of the genotype on behavioral lateralization. More precisely, we used a linear mixed effects model for repeated measures with the continuous variables direction “LI” or strength “|LI| ” of lateralization in session 1 as dependent variables and the fixed between-subject factors “genotype” (three levels: +/+, +/−, −/−) and “behavior” (four levels: GC, FRT, SG, BC), as well as their interaction. Furthermore, we included “batch” (two levels: 1, 2) and “animal ID” (*N* = 48) as the random between-subject factors.

#### 2.4.4. Temporal consistency of lateralized behaviors

For each behavior, we calculated a model to investigate whether the direction and strength of lateralization in session 2 is dependent on the same measure in session 1. Simultaneously, we checked whether interactive effects of the genotype with the direction and strength of lateralization in session 1 exist. For this, we used a linear mixed effects model with the continuous variables direction “LI” and strength “|LI| ” from session 2 as dependent variables and the continuous variables direction “LI” and strength “|LI| ” from session 1 and the factor “genotype” (three levels: +/+, +/−, −/−), as well as their interaction as fixed between-subject factors. Additionally, we included “batch” (two levels: 1, 2) as random between-subject factors. To further investigate the data, correlations between the two sessions were calculated using one-tailed Spearman’s rank correlations.

## 3. Results

### 3.1. Assessment of lateralization of behaviors

**FIGURE 3:**
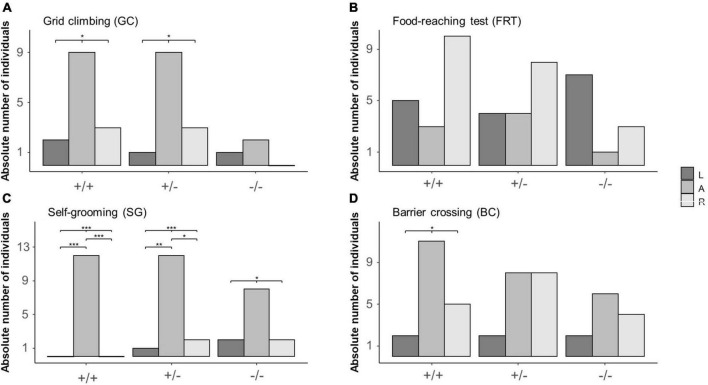
Distribution of left pawed, ambilateral and right pawed mice from three different 5-HTT genotypes across four different behaviors. Lateralized behavior of mice was observed during four different behaviors: **(A)** grid climbing (GC), **(B)** food-reaching (FRT), **(C)** self-grooming (SG), and **(D)** barrier crossing (BC). Based on individually calculated Z-scores, mice were categorized into being either left pawed, ambilateral or right pawed. Data are presented as absolute count frequencies. *P ≤ 0.05; **P ≤ 0.01; ***P ≤ 0.001. Significant results of chi^2^- and *post hoc* binomial tests are shown graphically.

During GC, the distribution of L, A and R mice differed from chance in 5-HTT +/+ (*L* = 2, *A* = 9, *R* = 3; χ^2^_2_ = 6.14, *P* = 0.046) and 5-HTT +/− (*L* = 1, *A* = 9, *R* = 3; χ^2^_2_ = 8, *P* = 0.018), but not in 5-HTT −/− mice (*L* = 1, *A* = 2, *R* = 0; χ^2^_2_ = 2, *P* = 0.368, see Figure 3A). However, after correcting for multiple testing, *post hoc* comparisons revealed no significant differences between the numbers of L, A and R mice in the 5-HTT +/+ and +/− genotypes.

In the FRT, the distribution of L, A, and R mice did not deviate from chance for all three genotypes (5-HTT+/+: *L* = 5, *A* = 3, *R* = 10; χ^2^_2_ = 4.33, *P* = 0.115; 5-HTT+/−: *L* = 4, *A* = 4, *R* = 8; χ^2^_2_ = 2, *P* = 0.368; 5-HTT−/: *L* = 7, *A* = 1, *R* = 3; χ^2^_2_ = 2, *P* = 0.078, see Figure 3B).

During SG, the distribution of L, A and R mice differed from chance in all three genotypes (5-HTT+/+: *L* = 0, *A* = 12, R = 0; χ^2^_2_ = 24, *P* < 0.001; 5-HTT+/−: *L* = 1, *A* = 12, *R* = 2; χ^2^_2_ = 14.8, *P* < 0.001; 5-HTT−/−: *L* = 2, *A* = 8, *R* = 2; χ^2^_2_ = 6, *P* = 0.050, see Figure 3C). Furthermore, in 5-HTT +/+ and 5-HTT +/− mice, there were less left pawed than ambilateral (binomial tests: 5-HTT +/+: *P* < 0.001; 5-HTT +/−: *P* = 0.010) and less right pawed than ambilateral mice (binomial tests: 5-HTT +/+: *P* < 0.001; 5-HTT +/−: *P* = 0.039).

During BC, the distribution of L, A and R mice differed from chance in 5-HTT +/+ mice (*L* = 2, *A* = 11, R = 5; χ^2^_2_ = 7, *P* = 0.030), but not in 5-HTT +/− (*L* = 2, *A* = 8, *R* = 8; χ^2^_2_ = 4, P = 0.135) and −/− mice (*L* = 2, *A* = 6, *R* = 4; χ^2^_2_ = 2, *P* = 0.368, see Figure 3D). However, after correcting for multiple testing, *post hoc* comparisons revealed no significant differences between the numbers of L, A, and R mice in the 5-HTT +/+ genotype.

### 3.2. Effect of genotype on direction and strength of lateralization

**FIGURE 4:**
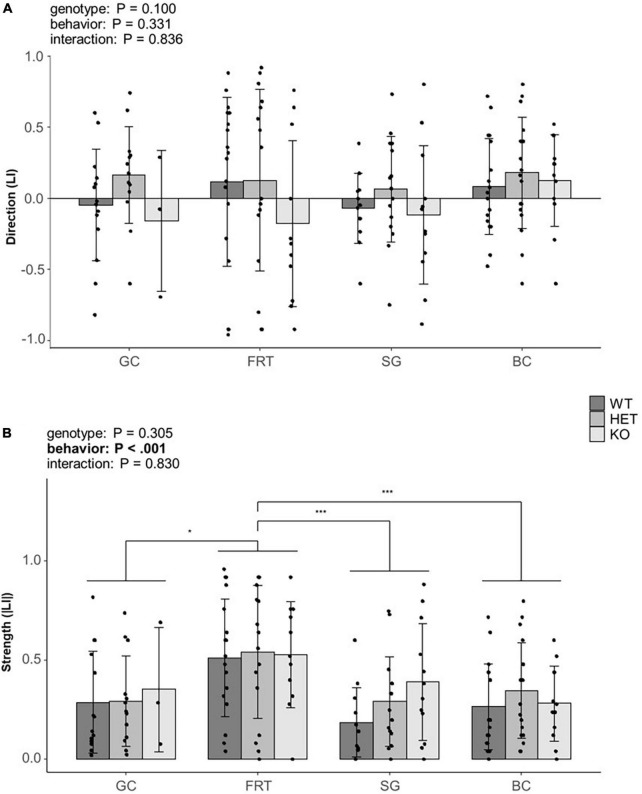
Influence of genotype on behavioral lateralization. **(A)** The direction (LI) and **(B)** the strength (|LI|) of lateralization of mice from three different genotypes (5-HTT +/+, 5-HTT +/– and 5-HTT −/−) for four different behaviors [grid climbing (GC), food-reaching (FRT), self-grooming (SG), and barrier crossing (BC)] was observed in two sessions (5 weeks apart). Statistics: Linear mixed model, *post hoc* Tukey HSD; Sample sizes: GC_+/+_ = 14, GC_+/–_ = 13, GC_–/–_ = 3, FRT_+/+_ = 18, FRT_+/–_ = 16, FRT_–/–_ = 11, SG_+/+_ = 12, SG_+/–_ = 15, SG_–/–_ = 12, BC_+/+_ = 18, BC_+/–_ = 18, BC_–/–_ = 12. Data is presented as means ± SD. *P ≤ 0.05; ***P ≤ 0.001. *P*-values of main effects are given as text. Significant *post hoc* effects are shown graphically. Behavior as main effect significantly affected the strength of lateralization.

The direction of lateralization (LI) was neither influenced by the genotype, the behavior nor by an interaction between both (Figure 4A, for statistical details see Supplementary Tables 1, 3), thus suggesting no effect of the behavior nor genotype on the direction of lateralization.

The strength of lateralization (|LI|) was influenced by the behavior [*F*(3,150) = 7.133, *P* < 0.001], indicating a behavior-specific lateralization. *Post hoc* comparisons revealed a stronger lateralization in the FRT compared to all other behaviors (GC: *P* = 0.029; SG: *P* < 0.001; BC: *P* = 0.001, see Figure 4B). However, neither genotype nor the interaction of genotype and behavior were statistically significant (for statistical details see Supplementary Tables 1, 3).

### 3.3. Temporal consistency of lateralized behaviors

**FIGURE 5:**
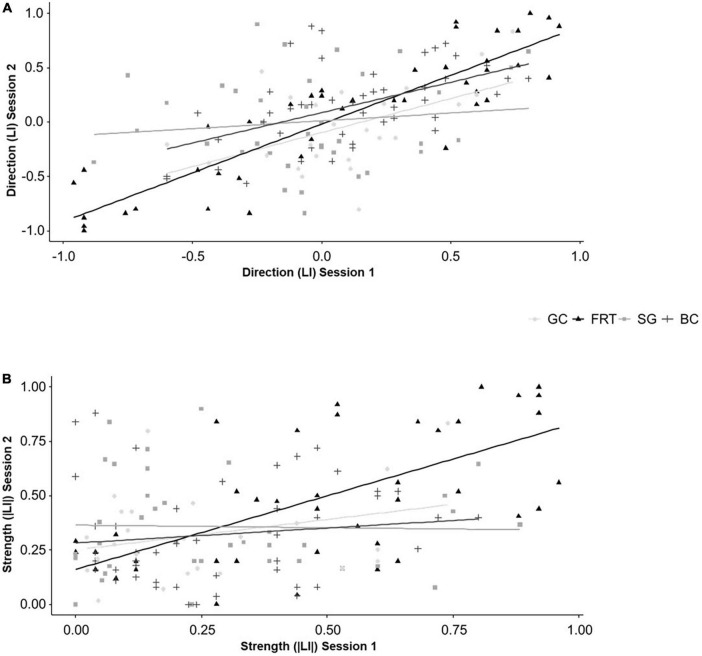
Temporal consistency of lateralized behaviors. **(A)** The direction (LI) and **(B)** the strength (|LI|) of lateralization of mice from three different genotypes (5-HTT +/+, 5-HTT +/− and 5-HTT −/−) for four different behaviors [grid climbing (GC), food-reaching (FRT), self-grooming (SG), and barrier crossing (BC)] was observed in two sessions (5 weeks apart). Because the genotype did not influence the temporal consistency, the data of all three genotypes was pooled for the graphical presentation. Statistics: Spearman correlations; Sample sizes: GC_LI = |LI|_ = 24, FRT_LI = |LI|_ = 43, SG_LI = |LI|_ = 37, BC_LI = |LI|_ = 47. Regarding the direction (LI), the FRT and BC were temporally consistent. Regarding the strength (|LI|), the FRT was temporally consistent.

The direction (LI) of lateralized behaviors in session 2 was influenced by the direction (LI) from session 1 in the FRT [*F*(1,36.023) = 154.200, *P* < 0.001] and for BC [*F*(1,41) = 20.923, P < 0.001], but not for the other two behaviors (for statistical details see Supplementary Tables 2, 4). Additionally, there were no interactive effects of genotype (for statistical details see Supplementary Tables 2, 4). Results of the Spearman correlations further illustrate the findings. After correcting for multiple testing, there are positive correlations between the direction (LI) from session 1 and 2 for the FRT (*r*_*s*_ = 0.890, *N* = 43, *P* < 0.001) and BC (*r*_*s*_ = 0.530, *N* = 47, *P* < 0.001, see Figure 5A).

The strength (|LI|) of lateralized behaviors in session 2 was influenced by the strength (|LI|) from session 1 in the FRT [*F*(1,36.988) = 29.330, *P* < 0.001], but not for the other behaviors (for statistical details see Supplementary Tables 2, 4). The strength of GC in session 2 was influenced by the interaction of the strength from session 1 and genotype [*F*(2,17.533) = 4.312, *P* = 0.030]. However, the Spearman correlations only partially support these findings. Regarding the FRT, there is a positive correlation between the strength from session 1 and 2 (*r*_*s*_ = 0.675, *N* = 43, *P* < 0.001, see Figure 5B). However, the differences in temporal consistency between the genotypes for GC disappeared after correcting for multiple testing (see Figure 5B and for statistical details Supplementary Tables 2, 4).

## 4. Discussion

In humans, non-right-handedness is associated with a higher incidence of psychiatric disorders (e.g., Markou et al., 2017; Ravichandran et al., 2017; however, see e.g., Packheiser et al., 2021). Since serotonin seems to be involved in both, the development of psychiatric disorders and lateralization, the present study investigated the effect of the serotonin transporter (5-HTT) on behavioral lateralization, using the 5-HTT knockout mouse model. We found no link between behavioral lateralization and 5-HTT genotype. However, the strength and temporal consistency of lateralization differed between the four behaviors observed.

### 4.1. No effect of 5-HTT genotype on behavioral lateralization

The 5-HTT genotype did not affect behavioral lateralization. Possibly, the behaviors observed in the study, *per se*, affected behavioral lateralization. More specifically, different specialized hemispheric functions (e.g., foraging and approach behavior, interaction with novel objects, emotion processing) (Rogers, 2002, 2009, 2010) are required to perform the behaviors. Since these functions are distributed between the two hemispheres, the respective hemisphere is activated when a particular function is required. Because hemispheric activity can affect behavioral lateralization (Rogers, 2009), inherent (in this case, 5-HTT-dependent) side preferences could thus be temporarily overridden. Another option is that the pleiotropic effect of the 5-HTT genotype extends to biochemical, anatomical and behavioral traits (Murphy and Lesch, 2008; Araragi and Lesch, 2013), but might not affect the mechanism underlying lateralization. For example, regarding biochemical traits, a reduction of 5-HTT’s (in 5-HTT +/− and −/− mice) increases extracellular serotonin content, most likely, equally throughout the brain and not in one hemisphere more than in the other. At least, no asymmetrical distribution of 5-HTT mRNA in the brain of mice was described (Bengel et al., 1997). However, since relative and not absolute neurotransmitter contents have been discussed to influence behavioral lateralization in rodents (Glick et al., 1977; Schwarting et al., 1987; Cabib et al., 1995; Nielsen et al., 1997) it is possible that an alteration in 5-HTT frequency does not affect behavioral lateralization as it does not affect neurotransmitter asymmetries (but see Mundorf et al., 2021). Yet another possibility is that the 5-HTT gene does not directly affect behavioral lateralization but rather affects susceptibility to environmental influences, which in turn could influence lateralization. In humans, carriers of a short allele are considered to be more susceptible to environmental influences, regardless of whether they are positive or negative (Belsky et al., 2009). In line, studies in 5-HTT knockout mice show that anxiety- and depression-like behavior was increased/decreased only after aversive/positive experiences (Carola et al., 2008; Jansen et al., 2010; Van den Hove et al., 2011; Kästner et al., 2015). The neutral housing conditions may have lacked opportunities for the animals to have impactful negative and positive experiences, which in turn, led to no changes in behavior and behavioral lateralization. Namely, pre- and post-natal stress, as a potential negative influence, has been suggested and shown to play a role in lateralization in humans and non-human animals (e.g., Fride and Weinstock, 1989; Johnson et al., 2018; for review see Ocklenburg et al., 2016) and the development of mental disorders (Berretz et al., 2020). Lastly, the link between behavioral lateralization and susceptibility to psychiatric disorders is probably more complex than just a monocausal relationship. For example, in humans, a recent study found an association between left-handedness and genes involved in the regulation of microtubules (Cuellar-Partida et al., 2021). Microtubules form part of the cytoskeleton, are important for several cellular processes and seem to play a role in neurodevelopmental disorders (Lasser et al., 2018). Thus, it is suggested that microtubule-mediated processes could link increased left-handedness with susceptibility to mental disorders (Cuellar-Partida et al., 2021). It is conceivable that similarly, complex pathways exist in non-human animals.

Nevertheless, descriptively, 5-HTT −/− mice preferred the left paw for reaching for food in the FRT, whereas 5-HTT +/+ and +/− mice preferred to use the right paw. In the light of lateralized emotional processing, this observation might be of interest. More precisely, our observation could suggest that left paw preference, thus right hemisphere dominance, in 5-HTT −/− mice is linked with negative emotion processing (Siniscalchi et al., 2021). This would be in line with the emotional valence hypothesis (Ahern and Schwartz, 1979; Silbermann and Weingartner, 1986; Heller et al., 1998). It would be interesting to investigate this further as it could be relevant for, for example, non-invasively assessing an individual’s emotional state and welfare (Rogers, 2010). However, it could well be that in the FRT, paw preferences independent from emotional processing are being measured (Simon et al., 2022). More precisely, the left hemisphere is generally involved in feeding behavior (Rogers, 2002). Since the FRT creates a “feeding/foraging context,” increased left hemispheric activation could affect paw preferences measured in the FRT. Notably, olfactory lateralization might be more promising for studying emotional processing in mice as it could be shown that mice use different nostrils for sniffing attractive versus aversive stimuli (Jozet-Alves et al., 2019).

### 4.2. Differences across the lateralized behaviors

Although there was no statistically significant effect of 5-HTT genotype, there were still some differences regarding the strength and temporal consistency of behavioral lateralization. Regarding the strength of lateralization, mice had stronger preferences in the FRT, compared to the other three behaviors. Notably, the FRT is a designed test for assessing paw use in a food-reaching task, whereas GC, SG, and BC are spontaneously displayed behaviors. There are mainly two hypothesis that can explain the observation of stronger preferences in more complex, forced behaviors, compared to less complex, more spontaneous behaviors. These two hypotheses are the “learning”- (Warren, 1980), and the “task-complexity”-hypothesis (Fagot and Vauclair, 1991), originally formulated on the basis of findings from studies with monkeys. The “learning”-hypothesis is supported by our results because it states that artificial test situations, i.e., the FRT, can induce and reinforce behavioral lateralization *via* learning, which has also been demonstrated in previous studies (e.g., Biddle and Eales, 2006; Ribeiro et al., 2011). The “task-complexity”-hypothesis assumes a population level lateralization for tasks that are more complex and cognitive demanding, i.e., the FRT. In line with a recent meta-analysis (Manns et al., 2021), we found only individual- but not population-level lateralization for the FRT (*n*_L_ = 16, *n*_R_ = 21). Thus, there is no indication that the “task-complexity”-hypothesis holds true, at least for mice.

The existing temporal consistency for the direction and strength in the FRT, and its absence for the direction and strength of the remaining spontaneous behaviors (except for the direction of BC) is in accordance with previous findings (Stieger et al., 2021) and can be explained by varying cost/benefit ratios of behavioral consistency. More precisely, to master the FRT, mice had to invest a high amount of energy to develop (i.e., learn) a behavior that enabled them to reach the food. Hence, because of the initial investment, the cost for changing this behavior is high. However, for spontaneous, less cognitive demanding behaviors, there was no initial, high energy investment and therefore, the costs of changing are smaller than the benefits. Additionally, individuals benefit from being better able to adapt to changes in the environment (Snell-Rood, 2013) and from being less predictable in specific contexts (e.g., prey-predator or competitive within-species interactions) (Frasnelli and Vallortigara, 2018).

In conclusion, since the 5-HTT genotype did not affect behavioral lateralization in mice, more research on other factors connected with behavioral lateralization and the development of symptoms of psychiatric disorders, such as environmental influences, is needed. For instance, future studies could incorporate experimentally controlled negative and positive experiences to induce the previously described phenotype in 5-HTT knockout mice. Additionally, lateralization of not only motor acts, but also sensory modalities, like olfaction as the predominant sensory modality in rodents, could be investigated.

## Data availability statement

The dataset presented in this study can be found in online repositories. The names of the repository/repositories and accession number(s) can be found below: https://doi.org/10.6084/m9.figshare.21458358.

## Ethics statement

The animal study was reviewed and approved by the local (Gesundheits- und Veterinäramt Münster, Nordrhein-Westfalen) and federal authorities (Landesamt für Natur, Umwelt und Verbraucherschutz Nordrhein-Westfalen “LANUV NRW”).

## Author contributions

SHR, NS, and SK conceived the study. SHR, NS, SK, and BS designed the experiments. SHR supervised the project. BS and YW carried out the experiments. BS conducted the statistical analysis of the data and wrote the initial draft of the manuscript. SHR, NS, SK, and YW revised the manuscript critically for important intellectual content. All authors contributed to the article and approved the submitted version.
