# Supplementary material for: Behavioral lateralization of mice varying in serotonin transporter genotype
Source: Front Behav Neurosci. 2023 Jan 11;16:1095567. doi: 10.3389/fnbeh.2022.1095567 (PMC9875089; doi:10.3389/fnbeh.2022.1095567)
Supplement: Supplementary file 1 [file Table_1.docx]

Supplementary Material

# Supplementary Tables

| **Supplementary Table S1. Statistical analysis of the influence of genotype and behavior on direction and strength of lateralization.**  (A) Presented are the effects of 5-HTT genotype (wild type (+/+), heterozygous (+/-) and homozygous knockout (-/-)) and behavior (grid climbing (GC), food-reaching (FRT), self-grooming (SG) and barrier crossing (BC) and their interaction on the direction (LI) and strength (\|LI\|) of lateralization (F-ratios, P-values, estimated effect sizes). (B) Tukey HSD post hoc comparisons. Data were transformed (Trans.) whenever deviating from normal distribution: SR = square root, NT = no transformation. P values in bold depict statistically significant differences (P ≤ 0.05). NumDF = numerator degrees of freedom, DenDF = denominator degrees of freedom, F = F ratio, P = P-value, η^2^p = partial eta squared (estimated effect size). |
| --- |
| **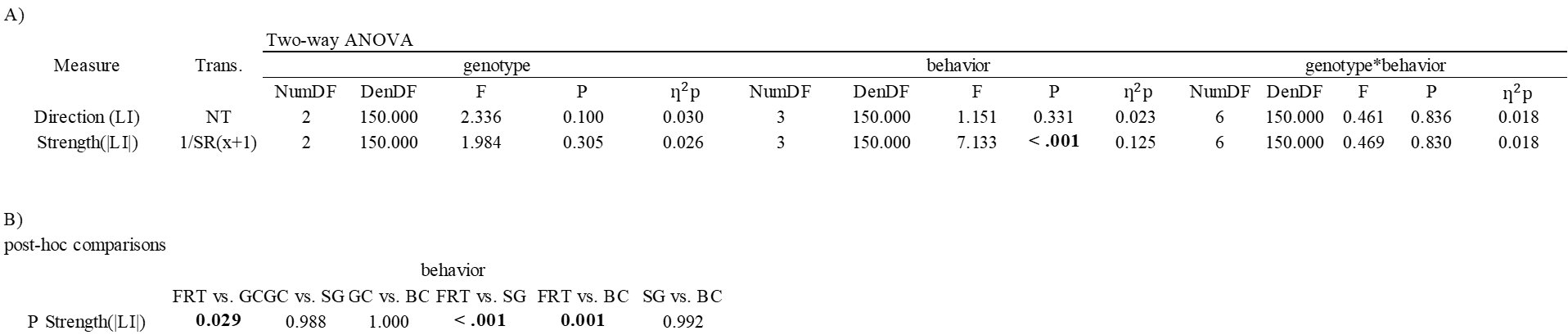** |

| **Supplementary Table S2. Statistical analysis of the influence of genotype and behavior on the temporal consistency of lateralization.**  Presented are the effects the laterality direction (LI) and strength (\|LI\|) of grid climbing (GC), food-reaching (FRT), self-grooming (SG) and barrier crossing (BC) in session 1 on LI and \|LI\| of these behaviors in session 2 (F-ratios, P-values, estimated effect sizes). 5-HTT genotype was included as an interactive effect. Statistics show the main effects of LI and \|LI\| in session 1, effects of interaction term of LI and \|LI\| in session 1 with 5-HTT genotype and Spearman correlations. Data were transformed (Trans.) whenever deviating from normal distribution: SR = square root, NT = no transformation. P values in bold depict statistically significant differences (P ≤ 0.05). NumDF = numerator degrees of freedom, DenDF = denominator degrees of freedom, F = F ratio, P = P-value, η^2^p = partial eta squared (estimated effect size), r_s_ = correlation coefficient. |
| --- |
| **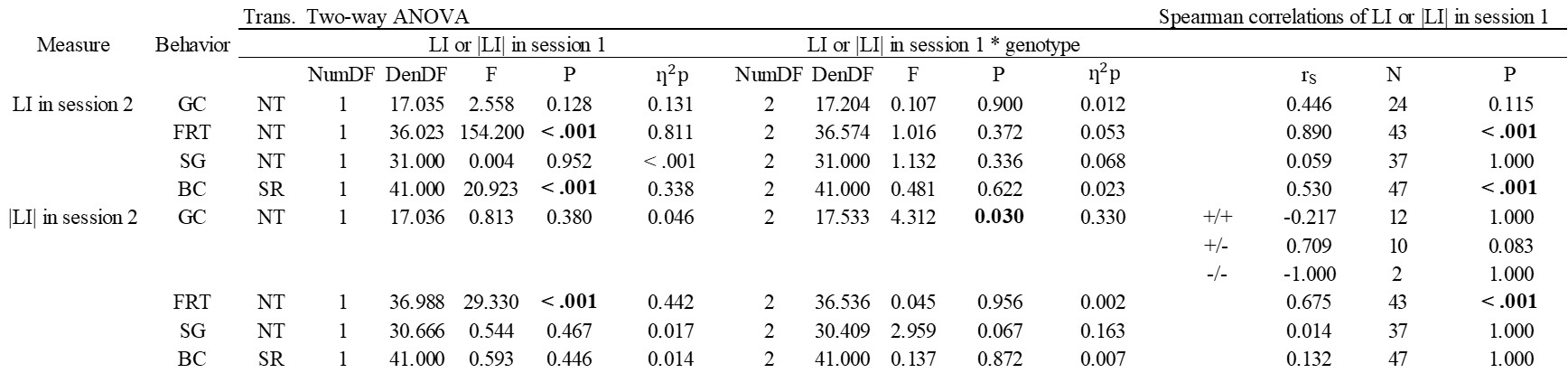** |

| **Supplementary Table S3. Data overview for the effects of genotype and behavior on direction and strength of lateralization.**  Summary of the effects of 5-HTT genotype (wild type (+/+), heterozygous (+/-) and homozygous knockout (-/-)) and behavior (grid climbing (GC), food-reaching (FRT), self-grooming (SG) and barrier crossing (BC) and their interaction on the direction (LI) and strength (\|LI\|) of lateralization. Data are presented as means ± SEM. P-values derive from linear mixed effects models with transformed data where necessary (see Supplementary Table S1 for more details). P-values in bold represents statistically significant differences (P ≤ 0.05). Sample sizes: N_+/+_ = 62, N_+/-_ = 62, N_-/-_ = 36, N_GC_ = 30, N_FRT_ = 45, N_SG_ = 39, N_BC_ = 48. |
| --- |
| 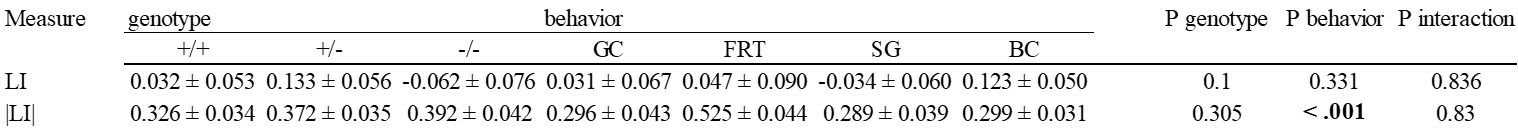 |

| **Supplementary Table S4. Data overview for the effects of the direction and strength of lateralization from Session 1 on lateralization from Session 2.**  Summary of the effects of direction (LI) and strength (\|LI\|) of lateralization from Session 2 and its interaction with 5-HTT genotype (wild type (+/+), heterozygous (+/-) and homozygous knockout (-/-)) on the direction (LI) and strength (\|LI\|) of lateralization from Session 1. Data are presented as means ± SEM. P-values derive from linear mixed effects models with transformed data where necessary (see Supplementary Table S2 for more details). P-values in bold represents statistically significant differences (P ≤ 0.05). Sample sizes: GC_+/+_ = 14, GC_+/-_ = 13, GC_-/-_ = 3, FRT_+/+_ = 18, FRT_+/-_ = 16, FRT_-/-_ = 11, SG_+/+_ = 12, SG_+/-_ = 15, SG_-/-_ = 12, BC_+/+_ = 18, BC_+/-_ = 18, BC_-/-_ = 12. |
| --- |
| 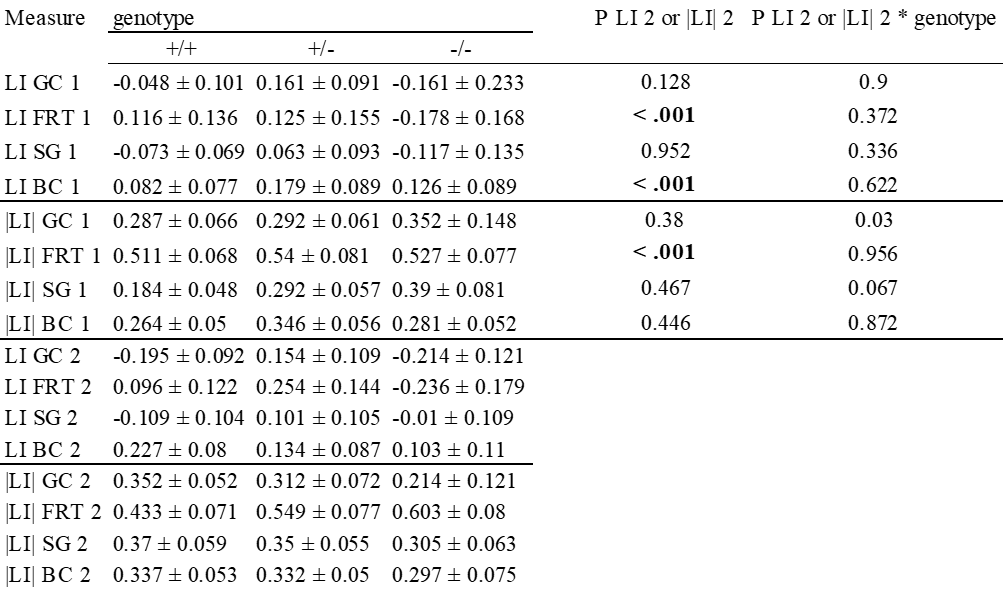 |
